# A hybrid method for X-ray optics simulation: combining geometric ray-tracing and wavefront propagation

**DOI:** 10.1107/S160057751400650X

**Published:** 2014-05-15

**Authors:** Xianbo Shi, Ruben Reininger, Manuel Sanchez del Rio, Lahsen Assoufid

**Affiliations:** a Advanced Photon Source, Argonne National Laboratory, Argonne, IL 60439, USA; b European Synchrotron Radiation Facility, 6 Rue Jules Horowitz, 38000 Grenoble, France

**Keywords:** hybrid method, beamline design, X-ray optics simulation, ray-tracing, wavefront propagation, partial coherence

## Abstract

A new method for beamline simulation combining ray-tracing and wavefront propagation is described. The ‘Hybrid Method’ computes diffraction effects when the beam is clipped by an aperture or mirror length and can also simulate the effect of figure errors in the optical elements when diffraction is present. The effect of different spatial frequencies of figure errors on the image is compared with *SHADOW* results pointing to the limitations of the latter. The code has been benchmarked against the multi-electron version of *SRW* in one dimension to show its validity in the case of fully, partially and non-coherent beams. The results demonstrate that the code is considerably faster than the multi-electron version of *SRW* and is therefore a useful tool for beamline design and optimization.

## Abstract 2

A new ’Hybrid Method’ combining ray-tracing and wavefront propagation for X-ray optics simulation is reported. The code is fast and can handle partially coherent sources, making it a useful tool for beamline design and optimization.

## 1. Introduction   

The accurate modeling of X-ray optics is essential for the design and optimization of synchrotron radiation beamlines, as well as for guiding the manufacture and characterization of optics. Many programs have been developed to simulate X-ray optics based on either geometrical ray-tracing or wavefront propagation. With the advent of high-brilliance synchrotron radiation sources with a high degree of coherence, simulation tools for describing beam from partially coherent sources and its propagation through various beamline optics and free space are in high demand. For example, the study of the effects of mirror figure errors on the beam properties (*e.g.* size, wavefront and coherence) is of great interest, especially for beamlines with diffraction-limited focusing mirrors.

Ray-tracing codes have been extensively used by the synchrotron radiation community for X-ray optics design and beamline optimization. The *SHADOW* code, written by Franco Cerrina in 1982 and first reported in 1984 (Cerrina, 1984 ▸), is by far the most popular ray-tracing tool developed for this purpose. The code is being continuously improved to incorporate new models and tools (Lai & Cerrina, 1986 ▸; Lai *et al.*, 1988 ▸, 1989 ▸; Sanchez del Rio *et al.*, 1992 ▸; Chen *et al.*, 1993 ▸, 1994 ▸). In 2011 it was upgraded to Fortran 95 using a modular approach (Sanchez del Rio *et al.*, 2011 ▸). Over the years, the *SHADOW* code has been widely freely distributed and used at synchrotron radiation facilitates worldwide (Brown & Hulbert, 1984 ▸; Reininger & Saile, 1990 ▸; Mythen *et al.*, 1992 ▸; Aksela *et al.*, 1992 ▸; Smith *et al.*, 1994 ▸; Reininger *et al.*, 1994 ▸; Zangrando *et al.*, 2001 ▸; Martins *et al.*, 2006 ▸; Songsiriritthigul *et al.*, 2007 ▸; Azuma *et al.*, 2010 ▸; Senba *et al.*, 2011 ▸; Alatas *et al.*, 2011 ▸; Sanchez del Rio & Alianelli, 2012 ▸; Chiuzbaian & Hague, 2012 ▸).

*RAY*, a ray-tracing code initially written by J. Feldhaus in 1984 (Feldhaus, 1984 ▸) and later upgraded by F. Schäfers and described in detail (Schäfers, 2008 ▸), has been used at a few facilities (see, for example, Mitzner *et al.*, 2005 ▸; Prasad *et al.*, 2010 ▸) besides BESSY (see, for example, Sawhney *et al.*, 1997 ▸; Weiss *et al.*, 1999 ▸; Gorovikov *et al.*, 2001 ▸; Könnecke *et al.*, 2013 ▸) where it was developed. Several other ray-tracing codes for simulating synchrotron radiation beamlines have been reported (Noda *et al.*, 1974 ▸; Yamada *et al.*, 2001 ▸; Knudsen *et al.*, 2011 ▸) but have not been widely used by the community.

The main limitation of ray-tracing codes, which are based on geometrical optics and intensity distribution, is in cases involving diffraction from an aperture, either a slit or a mirror, not accepting the full beam. This limitation was mostly encountered when simulating infrared, visible or vacuum-ultraviolet beamlines. The new low-emittance facilities (PETRA III), those under construction (NSLS-II, MAX IV, Sirius), or planned to significantly lower their emittance (ESRF, SPring-8, APS, ALS), are designed to incorporate beamlines for soft and hard X-rays where diffraction due to apertures or optical components have to be taken into account.

As opposed to ray-tracing, wavefront propagation codes are able to model the evolution of the synchrotron radiation emission generated by a single electron through the beamline optical elements and apertures. The propagation of the complex electric field in vacuum or through apertures is implemented in *SRW* (Chubar & Elleaume, 1998 ▸) in the frame of the scalar diffraction theory using Fourier optics methods. Until recently, optical elements were modeled in *SRW* assuming the thin-lens approximation. Elliptical cylinders, used in most beamlines in a Kirkpatrick–Baez (KB) configuration (Kirkpatrick & Baez, 1948 ▸), have been recently included in the code (Canestrari *et al.*, 2014 ▸). The implementation of additional optical surfaces in *SRW*, including gratings and crystals, is in progress.

*PHASE*, a wavefront-propagation code written by J. Bahrdt (Bahrdt, 1997 ▸; Bahrdt & Flechsig, 1997 ▸), is not limited to the small-angles approximation. *PHASE* is based on the stationary phase method and initially used a fourth-order Taylor series expansion to describe the image coordinates with respect to the source coordinates. Subsequently the code was extended (Bahrdt *et al.*, 2011 ▸) up to the seventh order in the coordinates and angles. A modified version of *PHASE*, based on ray-tracing and therefore avoiding the limitation of the number of terms in the Taylor series expansion, was used to simulate the bypass beamline at FLASH in Hamburg (Reininger *et al.*, 2001 ▸).

In general, the formalism of wavefront propagation only applies to coherent wavefields. To include the partial coherence of the synchrotron radiation source, theories and simulation tools have been developed based on coherent mode decomposition (Vartanyants & Singer, 2010 ▸; Singer & Vartanyants, 2011 ▸; Hua *et al.*, 2013 ▸) and Gaussian superposition (Idir *et al.*, 2011 ▸). On the other hand, geometrical ray-tracing codes like *SHADOW* assume that the X-ray source is either completely incoherent or coherent. The combination of wavefront propagation with ray-tracing is thus a natural choice for simulating partially coherent sources. It has been shown that wavefields whose coherent modes satisfy the Eikonal approximation could be treated within the framework of geometrical optics (Zysk *et al.*, 2005 ▸). The Monte Carlo Green’s function formalism (Fischer *et al.*, 2008 ▸; Prahl *et al.*, 2009 ▸) is also applied to X-ray optics by combining with ray-tracing (Prodi *et al.*, 2011 ▸). Recently, an analytical approach describing the propagation of partially coherent radiation through focusing optics has been developed (Singer & Vartanyants, 2014 ▸).

Among all available methods, the *SRW* code (Chubar *et al.*, 2010 ▸, 2011*a* ▸,*b* ▸, 2013 ▸) is by far the most developed program in simulating partially coherent synchrotron radiation. Electron beams in storage rings and linear accelerators are represented in *SRW* as micro-electrons distributed incoherently within the confined phase space. *SRW* then propagates the radiation from individual micro-electrons through the optical system and sums up intensities from all micro-electrons at a plane perpendicular to the optical axis. *SRW* is wave-optics-based and recently included local ray-tracing for simulating grazing-incidence focusing optics (Canestrari *et al.*, 2014 ▸). The method is straightforward and accurate; however, the multi-electron procedure required by simulating partially coherent sources is computationally expensive since it requires several hours to run in a multi-core environment. Furthermore, the wavefield evaluated at one plane cannot be directly carried over to another plane along the beamline. Any change in the optics parameters or the examination of the beam property at a new position requires performing, again, the full multi-electron simulation. Therefore, the code is not practical for beamline design and/or optimization.

We describe in §2 a new hybrid method combining ray-tracing with wavefront propagation, which offers a fast and efficient means to simulate and optimize synchrotron beamlines. The proposed code uses *SHADOW* as its backbone and extracts from its output files all the information required to perform wavefront propagation. The code seamlessly integrates diffraction effects due to beam clipping and the effects of figure errors of the optical elements.

The mirror figure errors are normally depicted in the spatial frequency domain, *e.g.* by the power spectral density function (Church *et al.*, 1979 ▸; Church, 1988 ▸; Church & Takacs, 1993 ▸; Siewert, 2008 ▸). In theory, the mirror surface perturbation for all frequencies could be described by the scalar theory (Beckmann & Spizzichino, 1963 ▸), of which the solution does not converge easily due to the infinite number of expansion terms (Aschenbach, 2005 ▸). In practice, the low spatial frequency components, which contribute to multiple foci, are mostly treated within the framework of geometrical optics (Sanchez del Rio & Marcelli, 1992 ▸; Signorato & Sanchez del Rio, 1997 ▸), while the high-frequency terms are evaluated by diffractive scattering under the smooth surface limit. The boundary between the two approaches is debatable and a recent work (Aschenbach, 2005 ▸) suggested that the geometric effect dominates when
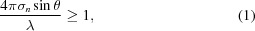
where *n* is an integer, 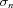 is the r.m.s. figure height error for the spacial frequency (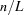) of a mirror with length *L*, θ is the grazing angle of the mirror, and λ is the X-ray wavelength. Otherwise, the wave effect dominates for the spatial frequency 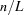 if the left-hand side of equation (1) is less than unity. As new low-emittance facilities are planned and built, ultra-smooth mirrors are required to produce diffraction-limited focus as well as to preserve the coherence of the X-ray beam. State-of-the-art mirrors with r.m.s. slope error below 0.05 µrad and r.m.s. height error below 1 nm (Siewert *et al.*, 2012 ▸) have already been manufactured. Based on equation (1), these state-of-the-art mirrors should be treated using the wave-optics approach even for low spatial frequencies.

Among the available wave-optics codes, mirror figure errors can be simulated on programs based on the stationary phase approximation up to a certain frequency (Bahrdt, 1997 ▸; Bahrdt *et al.*, 2011 ▸), over a broad spatial frequency range (Reininger *et al.*, 2001 ▸) or through the direct Fresnel–Kirchhoff diffraction integral (Yamauchi *et al.*, 2005 ▸; Kewish *et al.*, 2007*a* ▸,*b* ▸). The *SRW* code, based on Fourier optics, has been used to simulate figure errors in ideal optics (De Andrade *et al.*, 2011 ▸) and could also potentially cover the simulation of figure errors in its recent extension that includes grazing-incidence mirrors (Canestrari *et al.*, 2014 ▸).

In §3 we use the proposed hybrid method to study the effect of figure errors with different spatial frequencies on the focal spot. In §4 the capability of the hybrid method in dealing with partially coherent sources is benchmarked against the *SRW* code. The performance of the In-Situ Nanoprobe (ISN) beamline at the Advanced Photon Source (Maser *et al.*, 2013 ▸, 2014 ▸) is used as an example.

We note that the proposed hybrid method is adaptable to any ray-tracing code or wavefront-propagation algorithm. We have chosen the fast Fourier transform-based algorithm for its calculation speed and *SHADOW* for its popularity in the synchrotron radiation community. The integration of the hybrid code into *SHADOW* will be carried out in the near future.

## 2. Method description and mathematical procedure   

**Figure 1:**
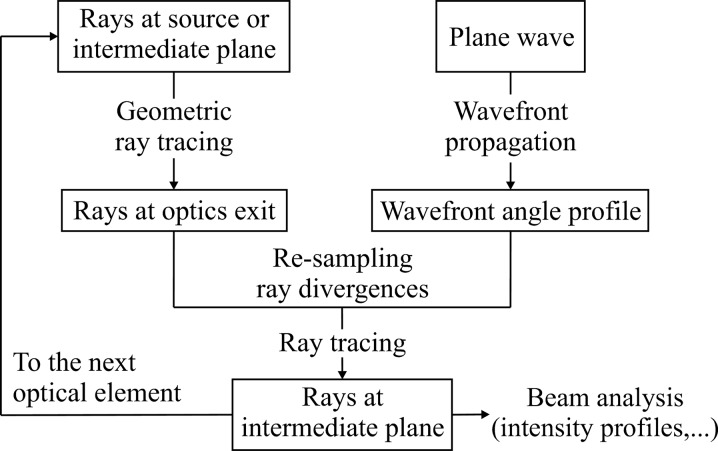
Flow chart describing the hybrid method combining ray-tracing and wavefront propagation.

The hybrid method simulates the geometric effects of the optical components (mirror figures) and apertures by means of ray-tracing, whereas their diffraction contributions are calculated independently for each element using wavefront propagation. The results from the diffraction contribution is integrated into the ray-tracing results by numerical convolution and ray re-sampling. Fig. 1 ▸ describes in a flow chart the procedure for a single element when the beam is being clipped by either an optical element or an aperture. Evidently, ray-tracing can be used when the beam is not clipped; and, in the case where the source is also diffraction-limited, the relevant distances are in the far-field. An entire beamline can be simulated by applying this procedure iteratively. The main calculation loop of the hybrid method is the ray-tracing part (left part of Fig. 1 ▸) since it provides most of the information required to perform the wavefront propagation when the beam is clipped.

In this paper the *SHADOW* code (Cerrina, 1984 ▸; Sanchez del Rio *et al.*, 2011 ▸) is used as the ray-tracing program and the reference for the coordinates definition. The spatial coordinates of the rays are defined in positions (*x*, *y*, *z*) and direction cosines (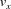, 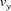, 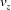). Ray divergences are given by d*x* = arctan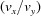 and d*z* = arctan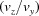. In the source plane or in the intermediate plane, the longitudinal axis *y* is the direction of the beam propagation. The transverse coordinates *x* and *z* are perpendicular to *y* and form the two-dimensional Cartesian coordinate system in the plane where *y* = constant. In the coordinates of the optics, the surface is defined in (*h*, *l*, *m*) with *h* along the surface normal, *l* tangential to the surface in the plane of (*h*, *y*), and *m* perpendicular to the plane of (*h*, *y*).

In order to include the diffraction effects calculated by wave optics into the ray-tracing procedure the ray-tracing is performed in two steps (Fig. 1 ▸). The first step starts from the source or the continuation plane, traces the element, and ends at the exit plane placed at zero distance downstream from the element (aperture or mirror). The rays limited by the aperture or mirror size are trimmed. Evidently, the ray-tracing takes into account the mirror figures (elliptical or parabolic) and generates the corresponding ray divergences (d*x*, d*z*). Therefore, the method is not limited to the thin-lens approximation. On the other hand, the diffraction effects of the optics will lead to additional deviation of ray divergences, which are not accounted for by ray-tracing. These effects, including the diffraction from the aperture or finite mirror size and from the figure errors deviating from the ideal elliptical or parabolic figures, are calculated by wavefront propagation. The results obtained from both ray-tracing and wavefront propagation are then combined together by the convolution of beam divergences, which is rationalized as follows. In the far-field approximation, the partially coherent beam incident on the optics can be viewed as a composition of plane waves with different propagation directions corresponding to the divergence distribution of geometric rays. Along each ray propagation direction, the plane wave diffracts through the optics and forms an angular distribution centered along that direction. One should also note that the convolution of beam divergences at the exit plane of the optics is equivalent to the convolution of beam profiles due to ray-tracing and wavefront propagation at the image plane. Since the convolution process is only a statistical approximation, the mutual interference effects are not included in the hybrid method.

The diffraction effect of the optics is evaluated by propagating a plane wave through an element using Fourier optics methods. The exit pupil function is defined according to the optical element. In the case of an aperture, the electric field in the coordinates of the exit plane 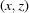 is expressed as
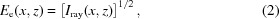
where the phase of the plane wave is chosen to be zero for simplicity. The amplitude of the wave is determined from the intensity distribution, 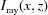, given by the ray-tracing. The range of the pupil function, determined by the aperture size or the size of the optic, is
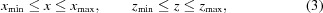
which is also obtained from the ray-tracing.

For optical elements, the intensity distribution 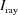 is not only apertured but also modified by the focusing (or defocusing) condition of the element. The use of the amplitude modulation 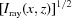 in the exit pupil function becomes essential to correct this change in the intensity distribution, especially for mirrors with figure errors.

The phase of the field 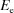 is modified by the figure errors of the optics (*i.e.* residual profile after the subtraction of the ideal elliptical or parabolic figure from the real mirror surface profile). By projecting the figure error profile from the mirror coordinates 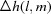 to the exit plane coordinates 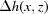, the phase shift 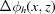 due to the mirror figure errors is given by
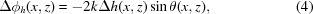
where *k* = 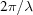 is the wavevector. Altogether, the electric field at the exit plane of the element is described mathematically as
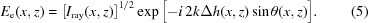
The wave boundaries (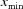, 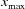, 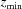, 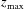) are again given by equation (3).

The angular profile of the wavefront intensity is calculated in the Fraunhofer diffraction approximation. The electric field 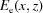 given by equation (2) (for an aperture) or equation (5) (for an optical element) is propagated in free space to the image plane at a large distance *y*, where the electric field is given by
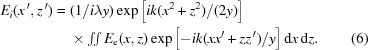
The discrete approximation of the Fourier transform in equation (6) can be carried out by using the fast Fourier transform algorithm. The angle intensity profile, or angle probability distribution function, at the exit plane is then given by
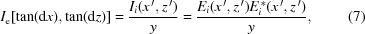
where the asterisk denotes the complex conjugate.

The procedure described above [equations (6) and (7)] is only valid in the far-field approximation (the Fraunhofer diffraction regime). In the case of an aperture of size Δ, the propagation distance *y* to the next optics should satisfy 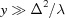 (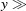 0.2 m, for instance for a 10 keV beam clipped by a 5 µm aperture). In the case of elliptical or parabolic focusing optics, the Fraunhoffer approximation is automatically satisfied at its focus.

The next step is to convolute the angle distribution from the wavefront propagation with the beam divergence from the ray-tracing at the exit plane of the optics. The convolution is achieved by re-sampling the divergences of all rays. For each ray, a random angle shift is generated based on the probability distribution function [equation (7)] and added to the divergence value retained from the ray-tracing. The summed divergence value is then used to obtain the direction cosines of the new ray. After such divergence re-sampling, the new rays contain both the effects of mirror figures and the diffractive contributions (*i.e.* from the aperture size and figure errors) of the optics. The ray positions obtained from ray-tracing are not changed at the exit plane of the optics.

Finally, the second ray-tracing is performed in free space to bring the new rays from the exit plane to the intermediate plane, where statistical analysis is carried out to acquire beam properties (*e.g.* total intensity and beam dimensions). The rays at the intermediate plane could be used as the source for the next optical element (*cf.* Fig. 1 ▸).

## 3. Simulation of a diffraction-limited focusing mirror   

The arrangement of two mirrors in a KB configuration (Kirkpatrick & Baez, 1948 ▸) is widely used to achieve small beam sizes. The two elliptical cylinder mirrors are orthogonal to each other and each mirror focuses the X-ray beam in a single direction. In most undulator-based beamlines, the beam footprint along the mirror sagittal direction, *m*, is relatively small, normally less than a few millimeters, and therefore it is not limited by the mirror width, as opposed to its size along the tangential direction *l*. Furthermore, due to the forgiveness multiplying factor ∼θ (DiGennaro *et al.*, 1988 ▸; de Castro & Reininger, 1991 ▸), the figure errors in the sagittal direction have relatively small effects on the X-ray beam. Therefore, the diffraction and slope errors of each mirror in the KB pair can be reduced to a one-dimensional simulation. In cases where the mirror focuses in both directions (*e.g.* toroidal and ellipsoidal mirrors), two-dimensional calculations may be necessary. Many open-source programs (*e.g.* *SRW*) are available for fast and robust two-dimensional Fourier optics simulations.

**Figure 2:**
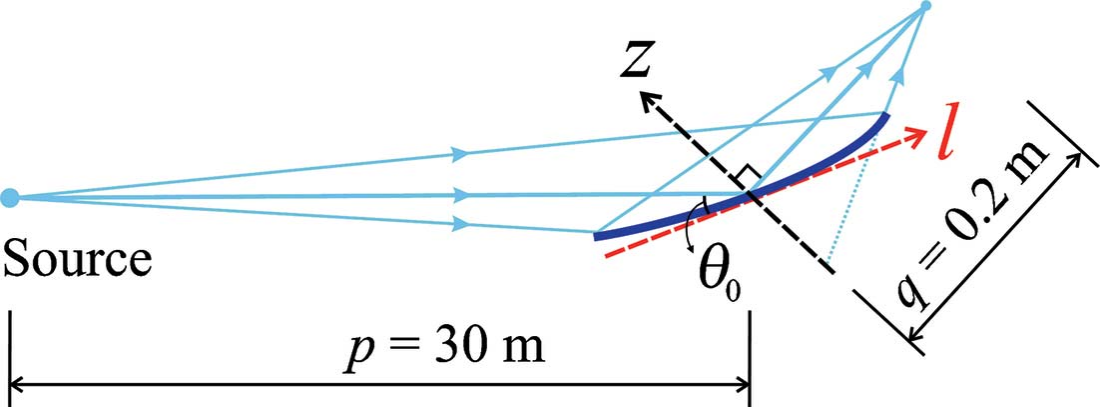
Schematic layout of the test case with an elliptical cylinder mirror focusing in the vertical direction.

In this paper, for demonstration purposes, we focus on the simulation of a diffraction-limited focusing mirror in one-dimension using the hybrid method. The optical layout and coordinate definition of the test case are shown in Fig. 2 ▸. The 10 keV (λ = 0.124 nm) X-ray source has Gaussian distributions in both size and divergence with r.m.s. values of 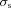 = 2 µm and 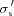 = 30 µrad, respectively. These values are chosen so that it mimics a secondary source generated from the five-to-one demagnification of the vertical source of the Advanced Photon Source (APS) (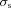 ≃ 10 µm and 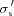 ≃ 6 µrad), which is not diffraction-limited since 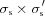 > 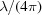. The source-to-mirror and mirror-to-image distances are *p* = 30 m and *q* = 0.2 m, respectively. The vertical focusing mirror is an elliptical cylinder of length *L* = 200 mm with grazing angle of 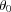 = 2.5 mrad at the mirror center.

**Figure 3:**
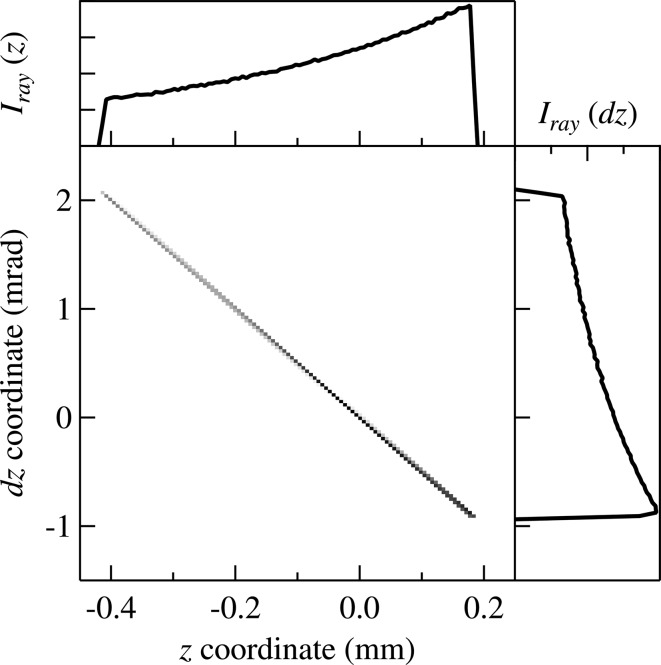
Phase-space image (left-bottom panel) of ray positions (*z*) and divergences (d*z*) at the optics exit (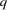 = 0) obtained from *SHADOW* ray-tracing. The intensity distribution profiles are plotted as a function of *z* (top panel) and d*z* (right panel).

Ray-tracing is performed through the optics (a single optical element in this case) yielding the beam intensity on a screen at zero distance downstream from the mirror. Ray-tracing results needed to calculate the pupil function are: (i) ray positions intercepting the mirror surface in coordinates 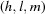, (ii) ray positions 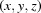 and directions (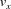, 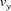, 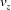) in the exit screen coordinates, and (iii) the incidence and reflection angles of each ray impinged on the mirror. Fig. 3 ▸ shows the phase space at the optics exit (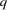 = 0) obtained from ray-tracing. The linear relationship between *z* and d*z* with a negative slope implies that the beam is converging due to the elliptical shape of the mirror.

The wavefront propagation relies on the description of the pupil function [equation (5)] with information evaluated from the ray-tracing results at 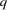 = 0. For the test case, we rewrite equation (5) as the one-dimensional expression
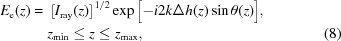
in the coordinate *z* shown in Fig. 2 ▸.

### 3.1. Without figure errors   

**Figure 4:**
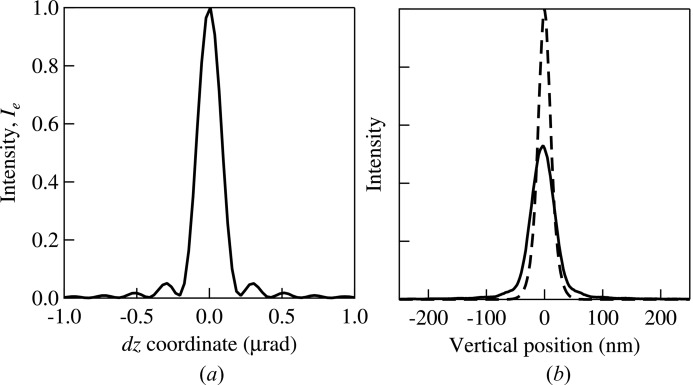
(*a*) Angular intensity profile 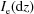 due to the mirror size at the optics exit (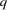 = 0) calculated using wavefront propagation. (*b*) Intensity profiles at the image plane (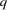 = 0.2 m) obtained by propagating the rays from the optics exit. Dashed line: calculated with the original ray divergences from the ray-tracing. Solid line: obtained by propagating the divergences convoluted with the wavefront propagation shown in (*a*). In both cases the ray positions at 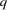 = 0 are those shown in Fig. 3 ▸.

When figure errors are not present, or 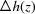 = 0, the exponential term in equation (8) reduces to unity. The amplitude of 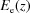 is obtained from the intensity distribution 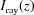 as the histogram of ray positions given in the top panel of Fig. 3 ▸. The boundaries of the exit wave are also determined from the boundaries of 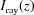 given by 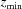 = −0.41 mm and 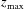 = 0.18 mm. Evidently, the effective aperture size 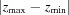 = 0.59 mm does not equal 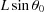 because the grazing angle is not constant along the mirror surface. The angular diffraction profile 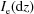 at 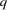 = 0 is calculated using equations (6) and (7) and shown in Fig. 4(*a*) ▸. The diffraction pattern is thus a result of the finite size of the mirror.

The angular distribution obtained from wavefront propagation [Fig. 4(*a*) ▸] is then convoluted with that from ray-tracing (the right-hand panel of Fig. 3 ▸) through the divergences re-sampling process described in §2.

The final step of the hybrid method involves the ray-tracing in free space from the plane 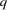 = 0 (with the convoluted ray divergences) to the image at 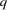 = 0.2 m. The intensity profile at 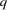 = 0.2 m is then extracted from the distribution of rays at this plane and it is shown by the solid line in Fig. 4(*b*) ▸. For comparison, the regular ray-tracing result obtained without including the diffraction effect is also shown in the figure by the dashed line. The difference in intensity and width between the regular ray-tracing and the hybrid method is obvious. Clearly, the hybrid method is necessary for simulating diffraction-limited cases.

### 3.2. With figure errors   

**Figure 5:**
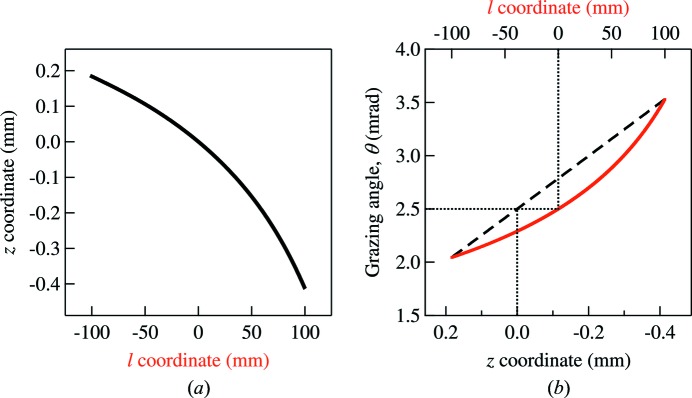
(*a*) Coordinate mapping from the mirror coordinate *l* to the exit plane coordinate *z* (see Fig. 2 ▸ for the coordinate definition). (*b*) Grazing angle θ in *l* coordinate (solid lines) and *z* coordinate (dashed lines). The dotted lines are a visual aid to show the mirror center.

When figure errors are present, the exponential term in equation (8) needs to be evaluated. The 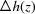 term is obtained by projecting the mirror figure error profile 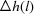 through the coordinate mapping 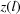 shown in Fig. 5(*a*) ▸. The coordinate mapping is extracted from a polynomial fit of *z* values in the exit plane as a function of *l* values on the mirror surface of all rays. Fig. 5(*b*) ▸ presents the grazing-angle variation as a function of the mirror coordinate 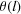 (solid line) and the exit plane coordinate 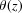 (dashed line). The grazing angle varies from 2 mrad to 3.5 mrad due to the proximity of the elliptical mirror to the image plane. Now, with all terms in the exit pupil function [equation (8)] fully described, the subsequent wavefront propagation, ray divergence re-sampling at the exit plane and ray-tracing to the image plane are performed in the same procedure as described in §3.1. Hereafter, we only focus on the results obtained from analyzing the ray statistics at the image plane.

**Figure 6:**
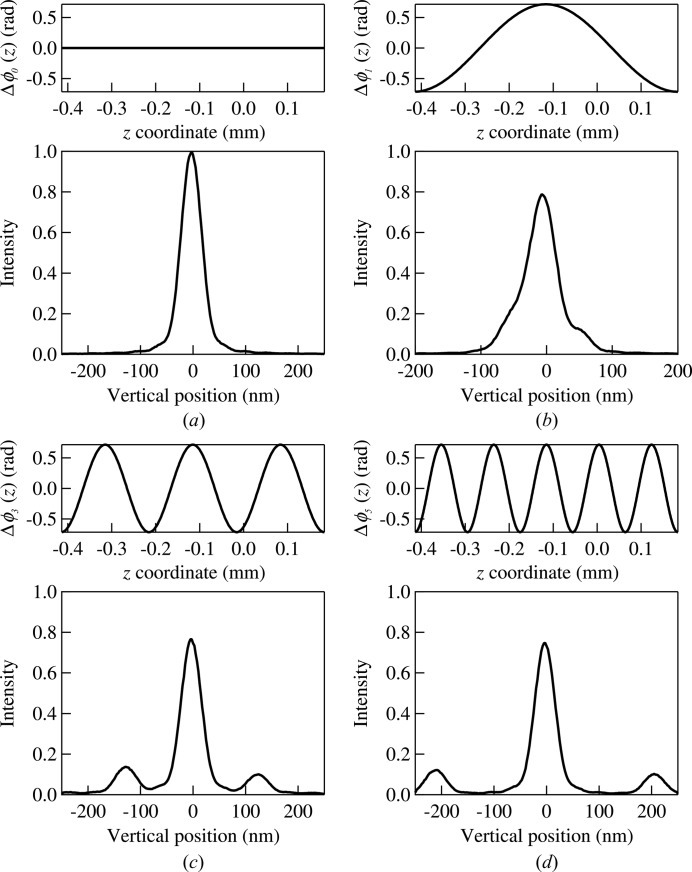
Intensity profiles (bottom figures) at the image plane (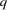 = 0.2 m) simulated with the hybrid method for single frequency figure errors with the corresponding phase shifts (top figures) given by equation (9) with (*a*) 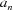 = 0, (*b*) *n* = 1, (*c*) *n* = 3 and (*d*) *n* = 5.

To illustrate the integration of figure errors into the hybrid method, we first calculate the effect of single spatial frequencies in the exit plane *z* coordinates. The phase shift 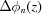 [the 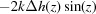 term in equation (8)] due to a single frequency that is a multiple, *n*, of (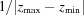) is described as
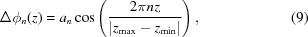
where 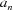 is the amplitude. The simulated intensity profiles using the hybrid method for *n* = 1, 3 and 5 with the same amplitudes (besides the 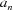 = 0 case) are shown in Fig. 6 ▸. All intensity profiles are normalized to the same area under the plot over the entire *z* range. In all figures, the distance between the positions of the first-order peak and that of the beam center is given by
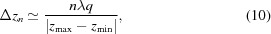
which is, in fact, the result given by a transmission grating. The low-frequency figure errors introduce additional peaks around the central peak, a mix of which effectively broadens the focus. As the frequency increases, the additional peaks are further away from the central peak and contribute to the scattering background, decreasing the intensity of the central peak without changing its shape.

**Figure 7:**
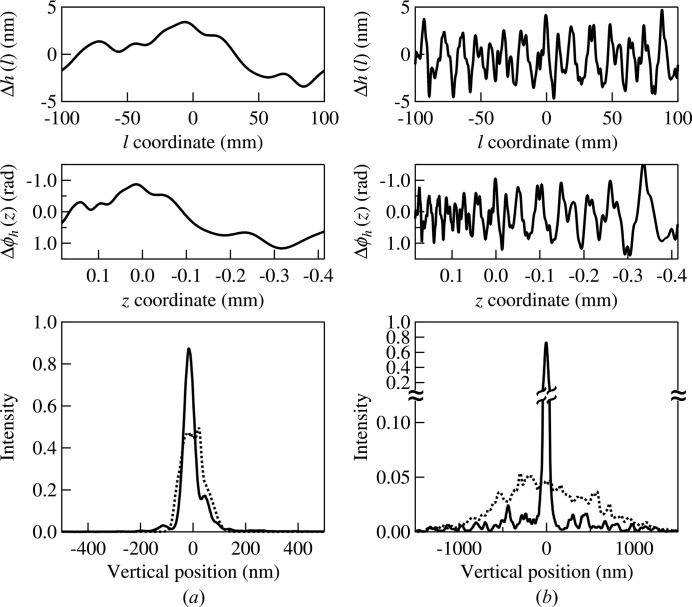
Top: figure errors generated with equation (11) for (*a*) *n* = 1–10 and (*b*) *n* = 11–100. Middle: phase shifts calculated using equation (4) from the above profiles. Bottom: intensity profiles at the image plane calculated using the hybrid method (solid lines) and *SHADOW* (dotted lines).

The figure-error profile of a mirror along its length in the mirror coordinate can be constructed by a series of sine or cosine components with frequencies that are multiples of (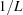) (Church & Takacs, 1993 ▸; Sanchez del Rio & Marcelli, 1992 ▸; Aschenbach, 2005 ▸), or
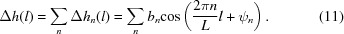
Because of the nonlinear coordinate mapping 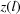 [*cf.* Fig. 5(*a*) ▸], a single frequency component in equation (11) is not directly related to that in equation (9). In the next example, we compare the results of the hybrid methods with those given by *SHADOW* for a mirror having either low-frequency figure-error components, *n* = 1–10, or high-frequency components, *n* = 11–100. Each individual frequency is generated based on equation (11) with 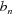 = 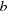 × 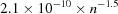 and a random phase 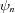. The value for 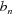 with 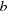 = 1 is an approximation to the measurements reported for a state-of-the-art mirror (Siewert *et al.*, 2012 ▸). The constructed figure error profiles for the low-frequency range and for the high-frequency range are shown in the top panels of Figs. 7(*a*) and 7(*b*) ▸, respectively. The r.m.s. figure error, 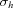, in each profile is set to 2 nm by scaling the corresponding partial sum with the scaling factor *b*. Since the r.m.s. slope errors 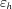 depend on the frequency range, its calculated value for the low-frequency profile is 0.1 µrad and its value for the high-frequency profile is 1.4 µrad. For demonstration purpose, the slope error value used here is very large to elucidate the effect of the high-frequency components. The corresponding phase shifts 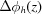 of the two profiles calculated using equation (4) are shown in the middle panels of Fig. 7 ▸. The nonlinear projections from 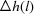 to 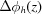 caused by the coordinate mapping given by Fig. 5(*a*) ▸ are clearly seen when comparing the top and middle figures. For both figure-error profiles, the left-hand side of equation (1) is less than unity for all frequencies, which indicates a stronger wave effect.

The solid lines in the bottom panels of Fig. 7 ▸ show the results of the hybrid calculations for the two profiles; the dotted lines show the results of the regular ray-tracing. All intensity profiles in Fig. 7 ▸ are normalized to the same area as in Fig. 6 ▸.

In the low-frequency case [Fig. 7(*a*) ▸], the intensity profiles given by the two methods have different shapes but the r.m.s. beam sizes are close. For the purpose of beamline design, both methods could provide reasonable results for guiding the mirror specification.

In the high-frequency case [Fig. 7(*b*) ▸], the two methods diverge completely. The width of the central peak in the hybrid case is similar to that of the ideal mirror case [*cf.* Fig. 6(*a*) ▸], but its peak intensity is reduced. Additional peaks spread across a large spatial range, mostly contributing to the scattering background. The r.m.s. beam width 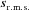 is related to the standard deviation of 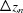 in equation (10) for all frequencies weighted by their magnitude 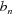. The FWHM of the beam is much smaller than 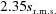 since the intensity clearly has a non-Gaussian distribution. On the other hand, the beam size obtained from the ray-tracing (dotted lines in Fig. 7 ▸) is approximately given by
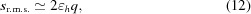
which does not consider the individual spatial frequencies.

The above results demonstrate that the hybrid method is more appropriate for simulating high-frequency figure errors. Since the hybrid method can handle different frequency ranges with either wavefront propagation or ray-tracing, it could be used to test the validity of different criteria, *e.g.* equation (1). The examples above were calculated using ‘synthetic’ figure errors based on equation (11). Metrology results combined with the hybrid method should provide a good estimate of the expected mirror performance.

## 4. Partially coherent beam   

The hybrid method can also provide a fast calculation of the statistical properties (*e.g.* intensity distributions) of a propagated beam, even when the source is partially coherent. In this section we benchmark this capability against the multi-electron *SRW* (the most developed code for simulating the effects of a partially coherent beam on a beamline), the single-electron *SRW* (for fully coherent beam propagation), and ray-tracing.

**Figure 8:**
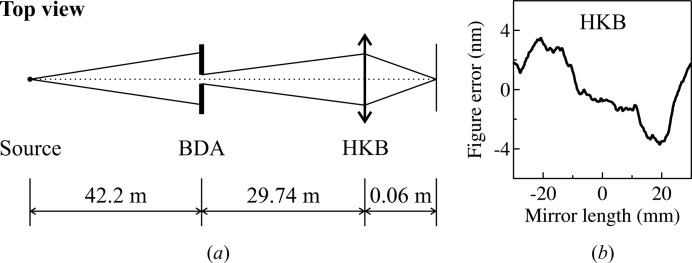
(*a*) Schematic layout of the In-Situ Nanoprobe beamline and (*b*) the figure-error profile of the horizontal KB mirror (HKB), which is constructed using equation (11) for 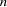 = 1–600 with an r.m.s. slope error of 0.5 µrad. BDA: beam-defining aperture.

The In-Situ Nanoprobe (ISN) beamline at the APS is used as the example for the benchmark. The beamline description and design parameters can be found elsewhere (Maser *et al.*, 2013 ▸, 2014 ▸). We choose the horizontal direction for the simulation [*cf.* Fig. 8(*a*) ▸] since the beam can be tuned from diffraction-limited (fully coherent) to partially coherent by adjusting the size of the beam-defining aperture (BDA). The source of this beamline is a 2.4 m-long APS undulator A with a period of 3.3 cm tuned to the first harmonic energy at 10 keV with the deflection parameter 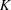 = 0.906. The electron beam parameters used are 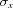 = 274.3 µm and 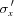 = 11.3 µrad.

The undulator source for the hybrid method (and *SHADOW*) used in this example is approximated at the center of the undulator by Gaussian distributions in size and divergence with r.m.s values given by (Elleaume, 2003 ▸)
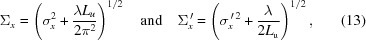
where 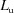 is the undulator length. The total horizontal beam size and divergence obtained from equation (13) are 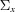 = 274.3 µm and 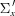 = 12.4 µrad.

In the single-electron *SRW* calculation, the single-electron emission (fully coherent) is obtained from the undulator parameters and propagated through the optical system. The single-electron *SRW* results, which contain no information about the electron beam, are shown in this paper for comparison only. The multi-electron *SRW* calculation sums up, at the image plane, the single-electron *SRW* results obtained from a large statistical sampling of the electron beam.

The horizontal KB mirror (HKB), which images the BDA at the sample position, is a 60 mm-long elliptical cylinder with a grazing angle 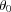 = 2.5 mrad at the mirror center. Fig. 8(*b*) ▸ shows the mirror figure-error profile used in the simulation, constructed using equation (11) for 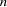 = 1–600 with a corresponding r.m.s. slope error of 0.5 µrad (for demonstration purpose, the value is chosen to be much larger than that of the state-of-the-art mirrors). The BDA and HKB are simulated as two individual optics in the hybrid method by applying twice the procedure described in Fig. 1 ▸.

**Figure 9:**
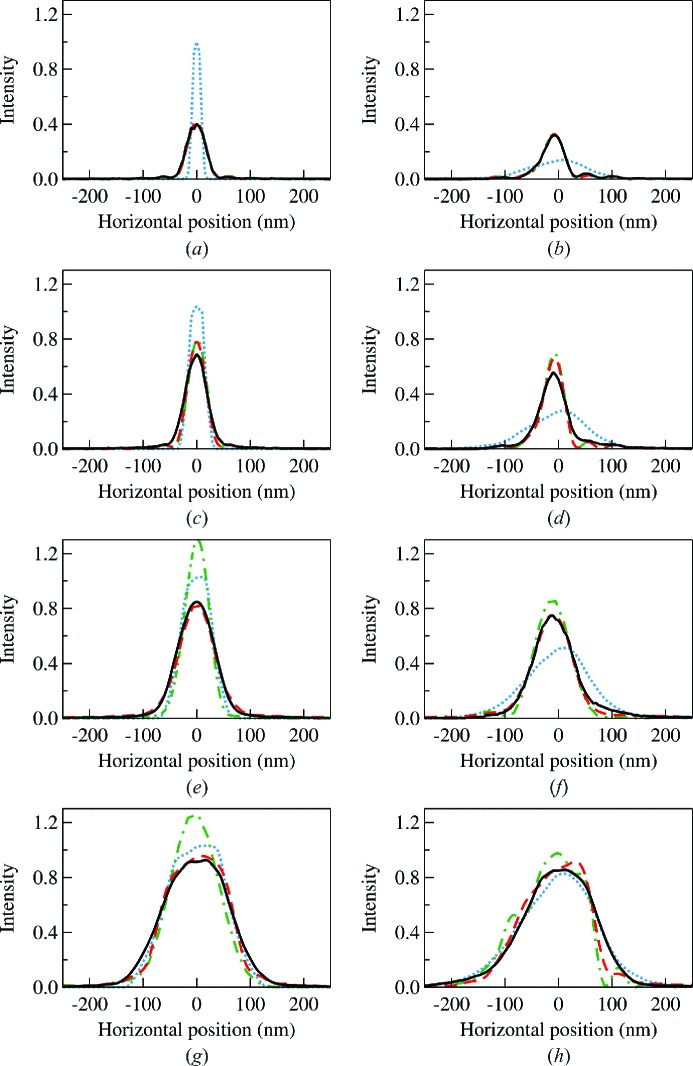
Horizontal intensity profiles of the ISN beamline simulated using the hybrid method (solid lines), the multi-electron *SRW* (dashed lines), the single-electron *SRW* (dash-dot lines) and ray-tracing by *SHADOW* (dotted lines) with different BDA sizes of (*a*, *b*) 10.5 µm, (*c*, *d*) 21 µm, (*e*, *f*) 42 µm and (*g*, *h*) 84 µm. (*a*), (*c*), (*e*) and (*g*) were calculated for an ideal elliptical cylinder mirror, while (*b*), (*d*), (*f*) and (*h*) also included the figure-error profile shown in Fig. 8 ▸(*b*).

Fig. 9 ▸ presents the simulation results using the hybrid method (solid lines), the multi-electron *SRW* (dashed lines), the single-electron *SRW* (dash-dot lines) and the *SHADOW* ray-tracing (dotted lines) with different BDA sizes. The figures on the right (left) are with (without) the figure errors given in Fig. 8(*b*) ▸. One should note that the figure-error calculation has not been fully implemented into *SRW* for grazing-incidence optics. Thus we treated the mirror figure error as an individual transmission element with the corresponding phase shift following the procedure described in this paper [*i.e.* equation (4)]. The intensity profiles in Fig. 9 ▸ are normalized to the total incident flux across the four methods. Since the horizontal source size is very large, the total beam intensity (area under the plot) is linear to the size of the BDA in all figures.

When the size of the BDA is limited to around 10 µm [*cf.* Fig. 9(*a*) ▸], the HKB is coherently illuminated (Maser *et al.*, 2014 ▸). Therefore, the beam size at the image plane is dominated by the diffraction effect. The pure ray-tracing calculation with *SHADOW* is not adequate because it excludes the diffraction from both the finite size of the BDA and the acceptance of HKB. The hybrid method provides near-identical results to that of both the single-electron and multi-electron *SRW* calculations in the fully coherent condition.

When the size of the BDA is 21 µm [*cf.* Fig. 9(*c*) ▸] there is a small difference between the hybrid method and the *SRW* results. The *SRW* cases clearly show the secondary maxima due to diffraction whereas in the hybrid method, due to the overestimation of the incoherence of the source by assuming a simple Gaussian distribution, the secondary maxima are less pronounced. However, the disagreement is not critical for beamline design purposes. The *SHADOW* result is again deviating largely from the other three methods.

At an aperture size of 42 µm [*cf.* Fig. 9(*e*) ▸], the agreement between the multi-electron and the hybrid mode is very good. As expected, the single-electron *SRW* starts to fail to describe the largely partially coherent source whereas the ray-tracing results start improving.

For a BDA of 84 µm [*cf.* Fig. 9(*g*) ▸], the beam can be considered completely incoherent and in the geometrical optics regime. The hybrid method and multi-electron *SRW* are alike and are very similar to the *SHADOW* results.

Similar comparisons are also shown in Figs. 9(*b*), 9(*d*), 9(*f*) and 9(*h*) ▸ when the mirror figure errors given in Fig. 8(*b*) ▸ are included. As discussed in the previous sections, the ray-tracing approach tends to overestimate the figure error effects from the high-frequency components, and therefore always provides larger beam sizes. In all cases, the hybrid method agrees well with the multi-electron *SRW* simulation.

The main advantage of the hybrid method is its fast computation speed. Each line in Fig. 9 ▸ takes only a few minutes on a single CPU processor in comparison with that of the multi-electron *SRW* simulation which takes several hours in a multi-processor environment. (The lack of a convergence-checking routine in *SRW* prevents us from providing the exact CPU time.) This feature makes the hybrid method an excellent tool for beamline design and optimization. Of course, the gain in speed is at the expense of losing the phase information of the beam due to the ray re-sampling process. As a result, it is not straightforward to extract from the hybrid method the mutual coherence function. However, some collective coherence properties could be retained within approximations; for example, the coherence length may be estimated from the intensity function of the effective source obtained by back ray-tracing. Further improvement of the hybrid method will include the implementation of better models to represent not only the source intensity distributions but also its coherence properties. The preservation of ray path length during the re-sampling is another challenge.

## 5. Conclusions   

In this paper, a hybrid simulation method combining the geometric ray-tracing and wavefront propagation is presented in detail. The figure-error effects on grazing-incidence mirrors are analyzed to perceive the boundary between the wave-optics and geometric optics limits. The hybrid method is compared with *SHADOW* and *SRW*, and is demonstrated to be accurate and fast for extracting the statistical properties of partially coherent synchrotron X-ray beamlines. Further investigations on the retrieval of beam coherence properties are being pursued. The hybrid method will be integrated into *SHADOW* so that its use could be transparent to the user.
